# DPPC Membrane Under Lateral Compression and Stretching to Extreme Limits: Phase Transitions and Rupture

**DOI:** 10.3390/membranes15060161

**Published:** 2025-05-26

**Authors:** Subhalaxmi Das, Nikos Ch. Karayiannis, Supriya Roy

**Affiliations:** 1 School of Applied Sciences, Kalinga Institute of Industrial Technology (KIIT) Deemed to be University, Bhubaneswar 751024, Odisha, India; das.subhalaxmi12@gmail.com; 2 Institute for Optoelectronic Systems and Microtechnology (ISOM) and Escuela Técnica Superior de Ingenieros Industriales (ETSII), Universidad Politécnica de Madrid (UPM), José Gutiérrez Abascal 2, E-28006 Madrid, Spain; n.karayiannis@upm.es

**Keywords:** molecular dynamics, phospholipid bilayer, biophysics, pressure, membrane rupture, atomistic simulation, DPPC, nematic order, compression, stretching, computational biophysics, membrane dynamics

## Abstract

Dipalmitoylphosphatidylcholine (DPPC), is one of the key bilayer membranes of the phosphatidylcholine (PC) family which constitutes 40–50% of total cellular phospholipids in mammal cells. We investigate the behavior of an initially planar DPPC membrane under lateral pressures from −200 to 150 bar at 323 K using microsecond-scale simulations. We identify, with very high precision, the pressure range for the occurrence of critical phenomena, mainly undulation and rupture. Notably, under compression, the membrane initially thickens, leading to a phase transition to an undulated state between 40 and 50 bar, as gauged by the sharp changes in the diverse structural metrics. Stretching induces systematic membrane thinning, with rupture becoming probable at −170 bar and certain at −200 bar. The reverse compression cycle shows pressure hysteresis with a 10-bar shift, while the reverse stretching cycle retraces the pathway. System size has a minimal impact on the observed trends. Under extreme mechanical stress, particularly near critical phenomena, simulation times on the order of microsecond are needed to accurately capture phase behavior and structural alterations. This work provides important insights into understanding membrane behavior under extreme conditions, which are relevant to numerous biological and technological applications.

## 1. Introduction

Cell membrane, primarily consisting of lipids and proteins, is a vital biological component that maintains the homeostatic environment inside the cell [1]. Lipids act as effective barriers controlling endocytosis and exocytosis while further serve as energy storage molecules [2]. In particular, cholesterol is crucial to membrane’s elasticity, fluidity, permeability, and structural integrity [3]. The physical, structural, and mechanical properties of membranes like fluidity and thickness indirectly regulate protein binding, enzymatic activity, gene expression, the efficacy of drug delivery, and the performance of the ion channels [4,5,6,7,8,9]. Many recent studies highlight the essential role of membranes in biology and provide insights on electroporation [10], amphiphile-membrane interactions [11], the complex dynamics of ions near membrane surface [12], and the elastic parameters in various systems and forms [13]. In the field of medicine, mechanical perturbation near the cell membrane, as magnetic nanoparticles under shear stress, induces magnetic fluid hyperthermia which can be an effective cancer treatment [14]. Nanoscale stress and mechanoporation damage in the POPC (1-palmitoyl-2-oleoyl-sn-glycero-3-phosphocholidonene) membrane during traumatic brain injury was studied by Murphy et al. [15]. A thorough understanding of how lipid membrane structure influences cellular processes potentially paves the way for biomechanically driven pharmacological therapies [16,17]. In many studies, cell membrane has been proven to be the origin of conversion of mechanochemical signals [18,19,20]. Mechanical stresses on biological cell membranes are converted into chemical or electrical signals that regulate various cellular functions like cell growth, signal transduction, and transport [21]. Interfacial electric fields play a key role in regulating membrane protein activity and ion movement across membranes, providing a basis for understanding how electrostatic interactions influence cellular processes [22]. Any mechanical disorder affects the static and dynamic properties of the membrane, including the thickness [23,24], structural order [25,26], polarity [27], viscosity [28], and fluidity [29]. Thus, it is essential to study the effect of external stimuli such as temperature, pressure, surface tension, and nano-inclusions on the mechanical and structural properties of cell membranes.

The recent developments in the field of molecular simulation have enabled it to complement theoretical and experimental attempts to tackle various complex scientific challenges [30]. Molecular Dynamics (MD) is one of the most widely used simulation tools to solve multi-body physical problems at the atomistic level. In the field of biophysics, MD has been widely used in understanding various physical phenomena such as the structural properties of lipid bilayers [31,32], protein–ligand binding [33,34,35], protein function [36] and design [37,38], binding of proteins on membranes [39,40], and drug–membrane interactions [41,42,43].

Recent research has elucidated the mechanical and dynamic behavior of lipid bilayers under varying stress conditions, providing insights into membrane fluidity, phase transitions, and stability. A study on cylindrically curved POPC membranes highlighted the role of membrane fluidity in facilitating deformation under stress, a critical factor in processes such as vesicle formation and membrane remodeling [44]. In DOPC bilayers, experiments and molecular dynamics (MD) simulations revealed that membrane tension modifies the dipole potential, influencing the electrostatic interactions with proteins or ions [45]. Kong et al. employed MD simulations and coarse-grained models to investigate surface tension-induced phase transitions in DPPC bilayers at 305 K. They demonstrated that changes in surface tension or temperature can drive transitions between liquid crystalline and gel phases in both pure and protein-embedded bilayers [46]. Similarly, Reddy et al. examined the impact of moderate tension on DOPC bilayers, far from the lysis thresholds, noting alterations in the physical properties that could affect membrane permeability or protein function [47]. Muddana et al. utilized atomistic simulations to study DiI-labeled DPPC bilayers across a range of area-per-lipid values (0.635–0.750 nm^2^), corresponding to surface tensions from −2.6 to 15.9 mN/m, underscoring the influence of lipid packing density on membrane mechanics [48]. At higher tensions, Cascales et al. reported a phase transition from a liquid crystalline to a gel phase in a 72-DPPC system at 330 K when surface tension reached ~40 mN/m [49]. Leontiadou et al. observed the formation of hydrophilic pores in DPPC bilayers, leading to rupture at ~90 mN/m (~−200 bar) [50]. Xie et al. corroborated these findings, noting DPPC membrane rupture at −200 bar under lateral stretching and undulation under 200 bar compression, irrespective of the pressure loading rate or membrane size [51]. Membrane undulation at 200 bar compression was also reported in the same study. Previous modeling studies suggested that systems under high compression may show very slow relaxation, requiring a very long simulation time to attain equilibration [46,52,53]. Also, the loading rate at which the pressure is applied is reported to affect the extent of conformational changes [51].

Hence, considering the slow relaxation and the effect of loading rates, the membrane behavior under extreme tension and compression is an attractive research topic, especially in identifying the critical conditions of significant structural alterations, in particular, undulation and rupture. Albeit the extended body of existing research, the structural and dynamical changes in a membrane during compression-induced undulation remain a topic which is not explored in sufficient detail. Here, we gauge, with unprecedented accuracy, the critical phenomena that occur in the phase behavior of the DPPC membrane and the corresponding pressures that trigger them. This includes a refined analysis of the uniform-to-undulated phase transition through distinct metrics. Phosphatidylcholine (PC) is the most prevalent phospholipid in mammalian cells, accounting for 40–50% of the total phospholipids in a cell [54]. The membrane under study, DPPC, is prominent among the PC family of membranes and is widely regarded as the hydrogen atom among the lipids [55]. It is a zwitterionic lipid that mimics the primary lipid component of eukaryotic membranes, and from a biological standpoint, it is regarded as a representative model for mammalian cell membranes [56].

Through microsecond-long atomistic MD simulations, exceeding by one order of magnitude any previous reported studies, we systematically gauge the effect of low and high loading rates, under lateral compression and stretching, on the structural characteristics and dynamical behavior of pure DPPC membranes. This allows us to further identify, with a very fine precision of 10 bar, the critical pressure conditions for undulation and rupture. The hysteresis behavior of the membrane is additionally studied by reverting the final undulated state down to normal conditions of 1 bar. Additionally, we systematically explore the effect of system size and the reproducibility of the present findings by conducting independent simulations under the same conditions. The paper is organized as follows: Section 2 presents the methodology; Section 3 hosts the results; and Section 4 concludes with a summary of the main findings.

## 2. Methodology

All MD simulations are performed with the open-source software suite Gromacs (version 2021.4) [57]. For all visualizations, we use the Visual Molecular Dynamics (VMD 1.9.3) software [58]. The main reference system corresponds to 256 DPPC molecules with 30 water molecules per DPPC, ensuring full hydration [59]. The initial configuration is created by replicating the original box from an initial structure of 64 DPPCs [60]. Additional simulations are conducted on a 128-DPPC system to identify the effect of the system size on the membrane behavior. Critical phenomena, like membrane rupture, are investigated by simulating four independent MD trajectories to check the reproducibility of the observed trends.

In all simulations we use the united-atom force field for DPPC derived by Berger, Edholm, and Jähnig [52]. The simple point charge (SPC) model is used for water [61]. All bonds are constrained to their equilibrium lengths with the linear constraint solver (LINCS) algorithm [62]. Periodic boundary conditions are applied to all axes. A cut-off distance of 1.2 nm is implemented for the Lennard–Jones interactions and the real space part of the electrostatic ones. The particle-mesh Ewald (PME) method [63] is used to compute long-range electrostatic interactions. For energy minimization, the steepest descent algorithm is implemented. All production MD simulations are performed in the isothermal–isobaric (NPT) ensemble with the Nose–Hoover thermostat [64,65,66]. Constant lateral pressure (along the XY-plane) is maintained using the semi-isotropic Parrinello–Rahman pressure coupling algorithm [67,68]. The integration time step is set to 2.0 fs and frames (system configurations) are saved every 100 ps. Depending on the conditions, to be discussed in detail later, the simulation time required to track effectively the critical phenomena reaches or even exceeds 1.5 µs, with multiple independent simulations performed to assess the reproducibility of the observed trends, totaling approximately 50 µs of simulations performed in the present study. Given that the computational cost of all-atom models is typically three to four times higher than that of the united-atom ones, making it impractical to achieve results on similar time scales, the united-atom model was selected for this study. Additionally, preliminary simulations on selected systems with the Berger united-atom and the CHARMM [69] explicit-atom force fields revealed no significant differences in the structural behavior when tested at 1 bar and 323 K. The area per lipid calculated using both force fields is very similar, averaging around 0.61 and 0.609 nm^2^ for LB and CHARMM, respectively. The average bilayer thicknesses for LB and CHARMM are found to be 3.8 and 4.1 nm, respectively, whereas the experimentally reported value is 3.9 nm [70]. The deuterium order parameter shows close agreements between these two forcefields. Additionally, the currently employed force field, which has been used in numerous studies over the years, including recent ones [10,12,71,72], shows very good to excellent agreement with the available experimental data, as will be explained in Section 3.

To study the change in the structural and mechanical properties of the membrane against lateral compression and stretching, pressure is varied in the range from +150 to −200 bar in a fine resolution of Δ*P* = 10 bar. The temperature is fixed at 323 K in all simulations. The pressure along bilayer normal, i.e., along the Z-axis, is fixed at 1 bar. It should be noted that negative pressure practically means tension. The target pressure is achieved through two different implementations, namely high loading (HL) and low loading (LL). For high loading, the target pressure is applied instantaneously to the final equilibrated configuration at *P* = 1 bar. For low loading, the target pressure is achieved progressively by ramping through intermediate steps of 10 and −10 bar for compression and tension, respectively, starting again from the same configuration (*P* = 1 bar). For LL at each intermediate step, a short NVT simulation of 1 ns is performed, followed by an NPT simulation of 30 ns, and the resulting configuration is used in the succeeding ramping step. Past studies [49] carried out the compression for just five surface tension couplings 0.6, 1, 6, 60, and 225 atm. A lateral tension study carried out in [51] observed the behavior of DPPC membrane for both high and low loading at 50, 100, and 200 bar.

**Figure 1:**
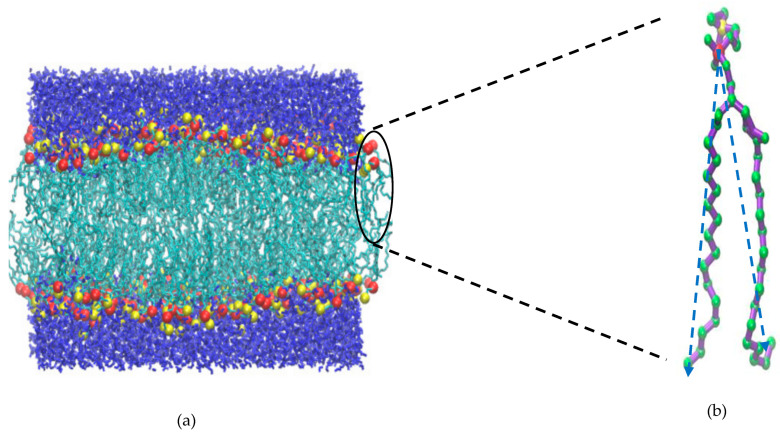
(**a**) Final configuration at the end of the NPT MD simulation of 200 ns on a system of 256 DPPC and 7712 water molecules at 1 bar. The red and yellow spheres represent the headgroup P and N atoms, respectively. The blue and cyan colors represent the solvent (water) and the acyl chains, respectively. (**b**) A single DPPC molecule in united-atom representation. Green color corresponds to methylene units. The nematic directors, the two vectors connecting atom P with the terminal carbon atoms of the two dangling acyl chains, are shown by blue dashed lines.

Initially, the system (256 DPPC and 7712 water molecules) is equilibrated for 200 ns at 1 bar and 323 K. A typical system configuration at the end of the simulation is shown in Figure 1. Below 50 bar compression, the simulation time is 400 and 600 ns for low and high loading, respectively. Whereas systems above 50 bar compression, are simulated for at least 1µs or even longer to achieve equilibration in HL compression. For stretching, the simulations for both loading protocols are carried out for 400 ns. In all cases, the simulation time is determined by the requirement to capture the possible structural transitions (undulation and nematic ordering) and critical phenomena (rupture). The main text focuses on the results obtained for low loading. The comparison between HL and LL is included in the Supplementary Materials as Figure S1.

### Area per Lipid 

In the present study, the area per lipid is characterized using two alternative definitions as follows: A_XY_PL and 3D-APL. A_XY_PL is defined as the average area occupied by a lipid molecule in the XY-plane of the membrane. A_XY_PL is calculated as (*L*_X_ · *L*_Y_)/*N*, where *N* is the number of lipids per leaflet and *L*_X_ and *L*_Y_ the box lengths in X and Y, respectively. Notably, A_XY_PL is essentially the area per lipid (APL) for a planar membrane; however, the latter becomes incorrect for an undulated membrane as curvature is introduced. To complement that, the three-dimensional area per lipid (3D-APL) is also computed using the Delaunay triangulation of the lipid headgroup positions, using the *Delaunay* module in the MDAnalysis [73,74] package to calculate the surface area of a 3D membrane. In this method, the XY-coordinates of the phosphorus atoms in the lipid headgroups are triangulated to estimate the three-dimensional surface area of each membrane leaflet, which is subsequently divided by the number of lipids to obtain the 3D-APL. This metric inherently accounts for local membrane geometry, providing an estimation of APL irrespective of the membrane geometry. It should be noted that membrane undulations could reduce the projected surface area (A_XY_PL) without necessarily affecting the intrinsic lipid packing density. The Delaunay triangulation approach addresses this by decoupling the geometric effects of undulations from the true changes in lipid packing, as it measures the surface area along the membrane plane rather than relying on its two-dimensional projection.

## 3. Results and Discussion

**Figure 2:**
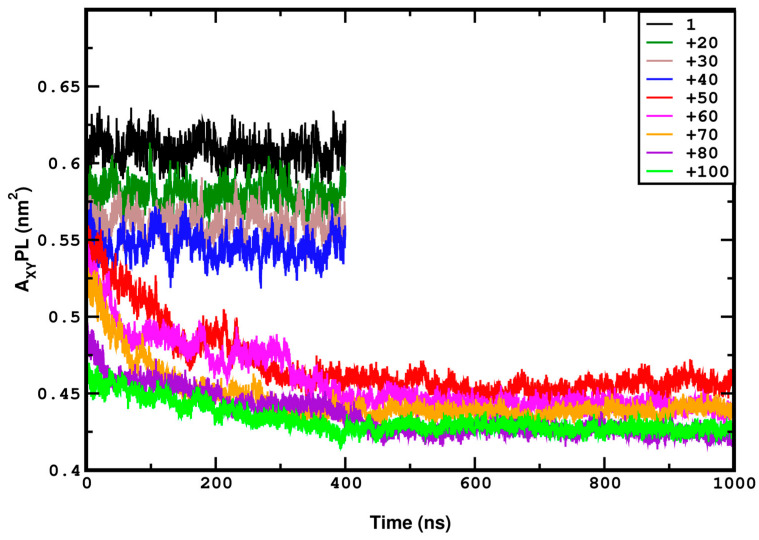
A_XY_PL versus the time taken for simulations corresponding to compression, demonstrating that systems subjected to high compression require extended time, nearly 400 to 500 ns, to reach equilibration. Here, the pressure is reported in bar, as in the rest of the figures.

To determine the time a system requires to reach equilibration, we study the time evolution of the XY projection of the area per lipid (A_XY_PL), as shown in Figure 2. As can be seen from Figure 2 at a pressure range at and above 50 bar, at least 500 ns are required for the system to reach the equilibrium state; hence, all corresponding simulations P≥50 have a duration that reaches or even exceeds 1 µs. We analyze the behavior of the last equilibrated part of the simulation trajectory (*t* > 500 ns). The systems at lower pressures, i.e., up to 40 bar, equilibrate within 30 ns. Their total simulation time (P < 50 bar) is 400 ns, out of which the last 350 ns are considered for the analysis.

### 3.1. Compression

**Figure 3:**
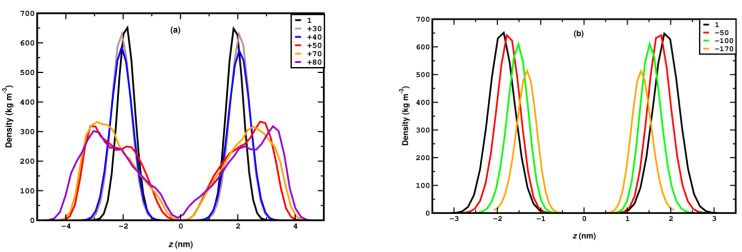

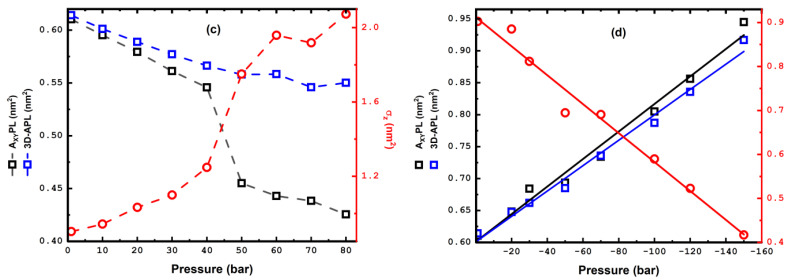
Mass density profile of DPPC headgroups along the membrane normal (Z-axis) for compression (panel (**a**)) and stretching (panel (**b**)). *z* (nm) represents the distance of the headgroups from the center. Panels (**c**,**d**) show the variation of A_XY_PL and 3D-APL (for the definitions see main text) against pressure for compression and stretching, respectively (left Y-axis), and the variance of the Z- coordinates of P-atoms, σ_Z_ is plotted against pressure (right Y-axis). The dashed lines connecting the simulation data in panel (**c**) serve as a guide for the eye. The solid lines in panel (**d**) correspond to best linear fits on the simulation data.

To illustrate the structural changes in the DPPC lipid bilayer with lateral compression, the mass density profile of the lipid headgroups along the bilayer normal (Z-axis) is plotted in Figure 3a for various pressures. The density profiles for up to 40 bar show two distinct peaks that correspond to the two well-defined positions of the lipid headgroups in the XY-planes in the top and bottom leaflets.

**Figure 4:**
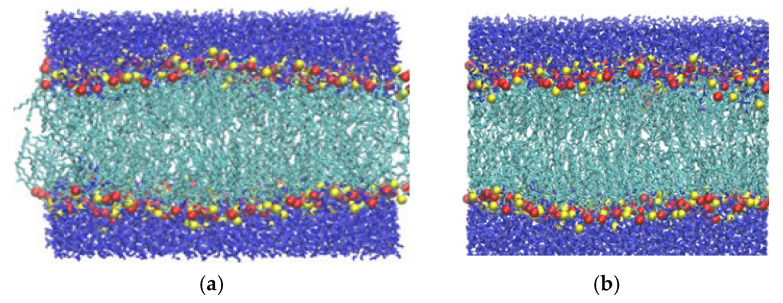

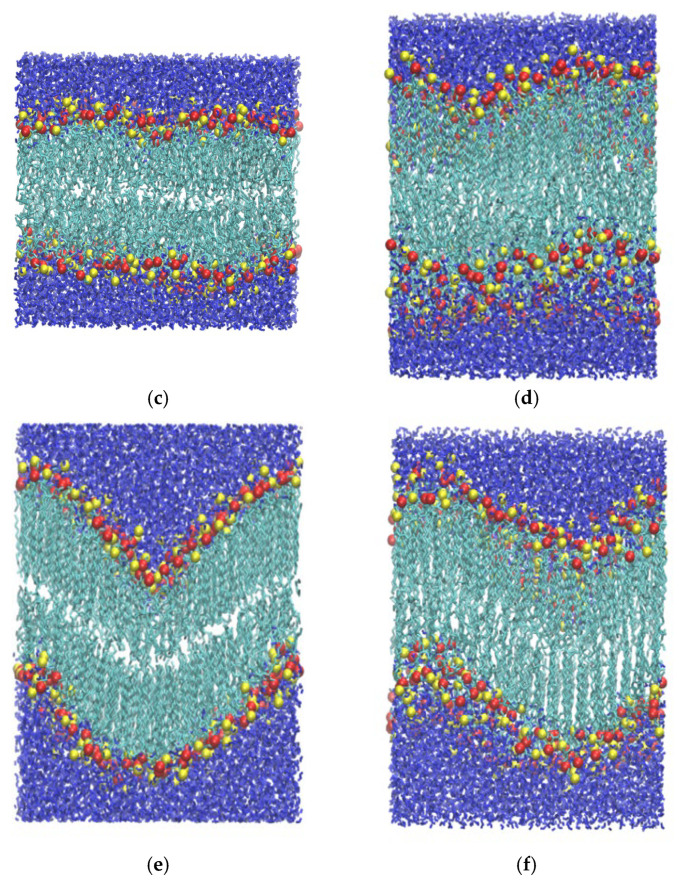
(**a**–**f**) Snapshots of the pure DPPC membrane during compression at 1, 20, 40, 50, 60, and 100 bar, respectively. The undulation of the membrane is distinctively observed at 50 bar and higher pressures. Color conventions and atom representations follow those of Figure 1.

The distribution of the headgroup remains very similar between 1 and 40 bar, except for a slight but regular increment in separation. This essentially means that the DPPC membrane retains its planar conformation for up to 40 bar of lateral pressure. This can also be confirmed by a visual inspection of the snapshots in Figure 4a–c. At 50 bar, an abrupt broadening of the peaks, accompanied by some irregularities, can be observed, a trend that evidently increases with pressure. The irregular broadening of the peaks can be attributed to the undulation of the membrane along the Z-axis, as shown in the snapshots in Figure 4d–f. This means that the membrane can withstand a lateral pressure of up to 40 bar without disrupting its planar arrangement, and it becomes undulated beyond that limit. The maximum lateral pressure applied to the system is 120 bar. At higher pressures, the membrane becomes laterally fused, which leads to the termination of the corresponding simulations.

In a previous study, Cascales et al. [49] performed 20 ns-long MD simulations using the GROMOS force field to examine the behavior of 72 DPPC molecules under lateral compressions of 0.6, 1, 6, 60, and 225 bar at 330 K. They observed a phase transition from liquid crystalline to gel at 225 bar but did not report a uniform-to-undulated phase transition, as observed in the present study. Their system withstood a lateral pressure of 225 bar, which differs significantly from our results; our system, subjected to +100 bar lateral pressure, was destroyed within 160 ns in the HL simulations. Although they used a different force field and a smaller system, the short simulation time of 20 ns could be the factor behind these differences. Our study indicates that systems under high compression require simulation times longer than 400 ns to reach equilibrium, as shown by the variation in the XY projection of area per lipid (A_XY_PL) in Figure 2.

**Figure 5:**
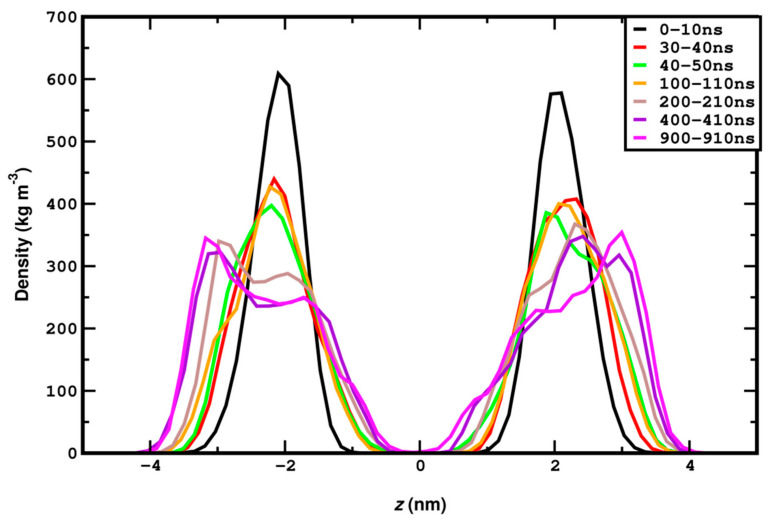
Mass density profile of DPPC headgroups along membrane normal (Z-axis) for compression (LL) with different time intervals along the simulation. An onset of undulations can be seen at around 100–110 ns.

The requirement for sufficiently long simulations also extends to gauge structural transitions and critical phenomena. The importance of sufficiently long simulation times for systems under compression can also be demonstrated by the time evolution of the mass density profile headgroups at 50 bar (Figure 5). The undulation is initiated at 40–50 ns and the shape of the curves changes significantly until 400 ns, after which it remains stable. It should also be noted that 400 ns is the minimum time required to equilibrate the highly compressed system, as shown by the data in Figure 2.

In Figure 3c, we compare the 3D-APL and A_XY_PL under both compression and stretching. At the standard condition of 1 bar, both metrics are found to have very similar values, approximately 0.61 nm^2^, which agrees very well with the experimental report of 0.62 ± 2 nm^2^ [75], as well as past simulation studies of 0.618 ± 0.3 nm^2^ [76]. Under compression, A_XY_PL initially decreases almost linearly and shows a sharp drop from approximately 0.55 nm^2^ at 40 bar (planar membrane) to 0.45 nm^2^ at 50 bar (undulated membrane); then, it decreases linearly at a slower rate. The 3D-APL also decreases linearly, though at a slightly slower rate compared to the A_XY_PL until 40 bar, corresponding to the planar membrane region. This decrease in 3D-APL is correlated to the increase in lipid packing density with the lateral compression. Beyond 40 bar, as the membrane undulates, unlike A_XY_PL, the 3D-APL does not show any sharp drop, rather, it exhibits a very slow irregular decrement in the undulated region. The absence of any sharp drop in 3D-APL across the transition indicates that the area per lipid is only weakly affected, implying no abrupt changes in lipid packing density. Consequently, the sharp drop in A_XY_PL is attributed primarily to geometric curvature effects rather than significant alterations in lipid packing. For lateral stretching, as shown in panel (d) of Figure 3, both A_XY_PL and 3D-APL increase linearly and closely follow each other. These increments suggest the gradual lowering of the lipid packing density, and consequently, the thinning of the membrane against stretching. For the pressure range from −150 to 40 bar, both the parameters adopt similar values, which again establishes that A_XY_PL is a suitable descriptor to estimate the APL of a membrane, provided the membrane remains in its planar conformation. Experimental [77,78,79] and simulation studies [80] on DPPC and DPPC/POPC mixtures have reported a sharp decrease in area per molecule during the transition from the liquid-expanded (LE) to the liquid-condensed (LC) phase, followed by membrane collapse under increasing surface pressure. This behavior resembles the phase transition and eventual membrane collapse under lateral stress observed in our study.

**Figure 6:**
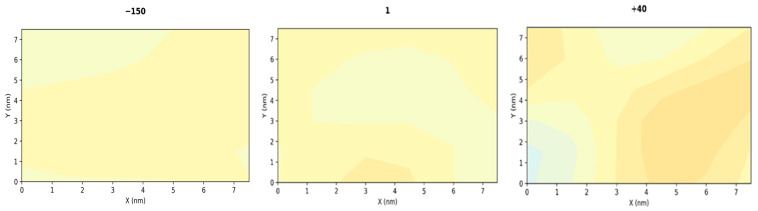

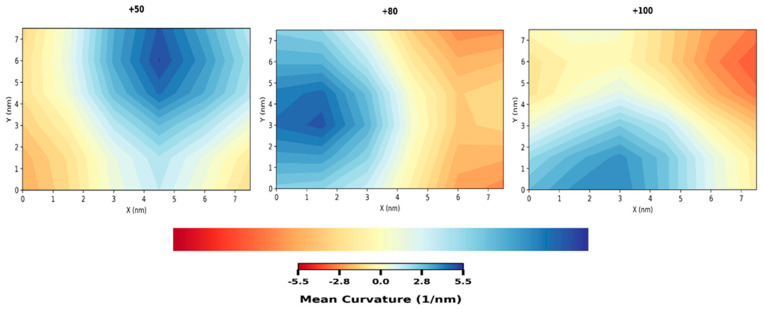
Contour plot of the mean curvature (nm^−1^) across the top leaflet leaflets of a DPPC 256 bilayer at −150, 1, 40, 50, 80, and 100 bar, projected onto the XY-plane. The 2D-projection contour plots use a three-color scheme as follows: blue, yellow, and red for convex, flat, and concave regions, respectively. The bottom leaflet shows very similar behavior, hence it is not presented here.

To illustrate the transition from a smooth to an undulated membrane, we further calculate the mean curvature across a range of lateral pressures. The mean curvature is computed using the phosphorus (P) atom of the DPPC headgroup to define the membrane surface for both leaflets using the *MembraneCurvature* module from the open source Python3 library MDAnalysis [73,74]. The resulting contour maps for membrane curvature are presented in Figure 6, where blue, yellow, and red colors correspond to convex, flat, and concave regions, respectively. At low pressures (1–40 bar), the yellow-dominated contours correspond to an almost flat or slightly concave surface. Beyond 50 bar, the emergence of deep blue and red regions signifies the development of highly convex and concave membrane patches, respectively, with curvature intensity increasing at higher pressures. This abrupt shift in curvature between 40 and 50 bar is consistent with the pressure-induced membrane undulation, observed in the present simulations, as gauged by independent measures (see Figure 3 and Figure 5). Under lateral stretching, the membrane surface retains an almost planar shape, as indicated by the low curvature values in the density maps for −150 bar. The contour maps for the intermediate pressure points are also included in the Supplementary Materials (Figure S2).

A previous study using a coarse-grained (CG) model reported that the application of pressure in both lateral and perpendicular directions led to a critical APL near the phase transition boundary of 0.57 nm^2^ [81]. The APL in the gel phase of DPPC was estimated at 0.465 nm^2^ and 0.46 nm^2^ using CG simulations [53] and experiments [59], respectively. However, in the present study, the 3D-APL decreases only slightly in the undulated phase (P > 40 bar), exhibiting a value in the range from 0.57 nm^2^ to 0.55 nm^2^. Higher APL corresponds to lower lipid packing density in the undulated phase compared to the gel one. Interestingly, both transitions occur at an APL value close to 0.57 nm^2^, which appears to be a critical threshold for the stable liquid crystalline phase in the planar membrane conformation. To characterize the membrane undulation, we measure the variance in the Z-coordinate of the phosphorus atoms in the headgroups, denoted as σ_Z_, for both the top and bottom leaflets. σ_Z_ is then averaged across multiple configurations. As depicted in Figure 3c, the relationship between *σ*_Z_ and lateral pressure initially exhibits a linear increase up to 40 bar, followed by a pronounced sharp increase between 40 and 50 bar. This abrupt increase is attributed to the significant membrane deformation, as illustrated in the snapshots in Figure 4. Additionally, the sharp change in σ_Z_ closely mirrors the trends observed in the density plot of the head groups (Figure 3a), the variation in the area per lipid (A_XY_PL) (Figure 3c), and in the curvature (Figure 6). This correspondence reinforces the association of the phase transition with enhanced membrane deformation.

**Figure 7:**
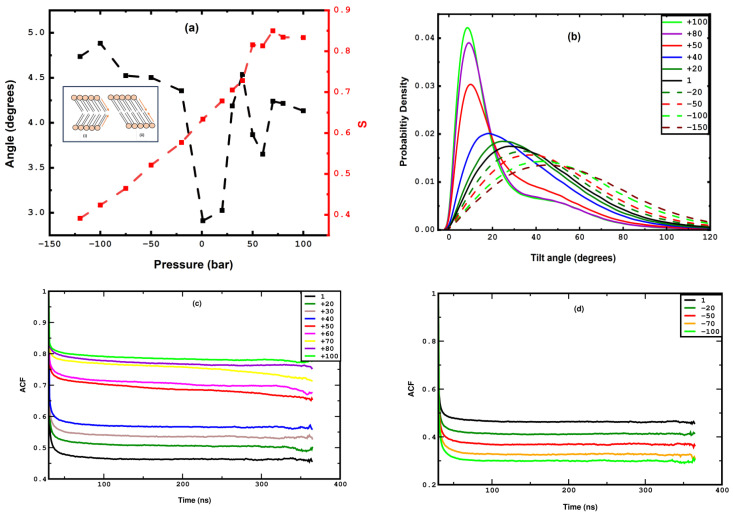
(**a**) Inset: A coarse-grained sketch of the DPPC layer is depicted. The orange circle represents the headgroup, and the straight lines represent the acyl chains. The orange arrows correspond to the global nematic directors of the top and bottom leaflet. Configurations (i) and (ii) correspond to the cross-tilt and tilt phases, respectively. Main panel: Global nematic order parameter, *S*, (shown in red, right Y-axis) and angular separation of the leaflet nematic vectors (shown in black, left Y-axis) as a function of pressure. (**b**) Distribution of tilt angles for alternate carbon atom vectors in both acyl chains of the DPPC molecules at different positive and negative pressures. (**c**,**d**) The (orientational) autocorrelation function, ACF (see Section 3.1 for definition), is plotted against time for compression (panel (**c**)) and stretching (panel (**d**)).

To quantify the effect of lateral pressure on the alignment of the lipid acyl chains, we further calculate the nematic order parameter for the acyl chain vectors for both leaflets, and we then average them to obtain the mean nematic order parameter, *S*. For the calculation of the nematic, long-range order parameter we follow the approach of [82,83,84]. Most importantly, this approach does not require the reference of the bilayer normal, rendering the metric valid for undulated membranes too. First, we consider the vectors that correspond to the two acyl chains of the DPPC molecule and connect the P atom of the headgroup with the terminal carbon atoms of each acyl chain, as illustrated in Figure 1b (and in more detail in the sketch of Figure S3). Based on this, we also calculate the global nematic vectors for the top and bottom leaflets, as illustrated in the inset of Figure 7a, a sketch like the one already reported in [85]. Given these two global vectors, we further calculate the corresponding angular separation between them. A value of angular separation close to zero indicates parallel alignment between the nematic directors of the two leaflets, suggesting a disordered or tilt phase. Nonzero, large angular separation indicates two different orientations of the nematic directors, hence corresponding to a cross-tilt phase. This allows us to differentiate between the tilt and cross-tilt phases, as reported previously in [85]. The results on the global nematic order parameter and the angular separation of the nematic vectors corresponding to each leaflet are shown in Figure 7a. The nematic order parameter increases almost linearly from 1 to 40 bar, indicating an increase in the lipid chain ordering due to lateral compression, and it exhibits a sharp jump between 40 and 50 bar, which corresponds to the phase transition. Beyond 50 bar, it reaches a plateau value in the undulated phase where no further increase is observed. This plateau behavior is due to the combined effect of the presence of high-ordered local domains and the slight loss of global ordering due to undulation. In parallel, the angular separation between the leaflet nematic vectors fluctuates randomly between 2 and 5 degrees for the entire range, exhibiting the lowest value at 1 bar. Since the variation is very small and the angle of the vector orientation between the leaflets adopts very small values, close to zero, we can conclude that a cross-tilt phase is absent for the whole pressure range.

To elucidate the structural and dynamical changes in the acyl chain segments, vectors connecting alternate carbons of the acyl chains are defined, and their tilt angles relative to the plane perpendicular to the XY-plane are analyzed (see the corresponding sketch in Figure S3e of Supplementary Materials), like in the study carried out by Leekumjorn and Sum [85]. The tilt angle distribution across a pressure range from 1 to 40 bar demonstrates a broad spectrum, reflecting the high flexibility of the alternating carbon segments. The peak of the tilt angle distribution for the 1 bar system is in close agreement with previous simulation studies [86,87]. As pressure increases, this distribution narrows progressively, and the peak shifts towards smaller tilt angles, indicating a tendency for these segments to align more closely with the bilayer normal. Notably, between 40 and 50 bar, the tilt angle distribution exhibits a distinct transformation as follows: it reveals a sharp maximum at approximately 10 degrees and a secondary, less pronounced peak, near 45 degrees. At pressures exceeding 50 bar, the primary peak becomes narrower, and the secondary one becomes more pronounced, signifying an enhanced structural order and a shift in the dynamics of the acyl chain segments under higher pressure conditions. The small plateau around 45 degrees in the tilt angle distribution in the undulated phases indicates the possible presence of the smaller disordered domains, as shown in Figure S3d in the Supplementary Materials. These system conformations, characterized by a predominantly ordered structure with some disordered domains, are referred to as the ripple phase, which is observed in the gel phase, as documented in [88]. Snapshots allowing for a visual inspection of the ripple phase are provided in Figure S4 (Supplementary Materials). The deuterium order parameter along the Z-axis is also calculated and presented in Figure S5 (Supplementary Materials), revealing the enhanced ordering of the acyl chains with an increase in compression.

The orientational segmental dynamics of the acyl chains is investigated by studying the autocorrelation function (ACF) of the vector between the alternate carbon atoms, as defined in the previous section. The autocorrelation function, *f*, is defined as

$$f(\delta t)=\langle \hat{n}(t)\cdot \hat{n}(t+\delta t)\rangle$$

where $\hat{n}(t)$ and $\hat{n}(t+\delta t)$ represent the unit vectors along alternating carbon atoms corresponding to time t and t+δt, respectively. The autocorrelation function, ACF, is averaged over all the alternate carbon vectors and multiple time origins. In Figure 7c, the ACF is plotted against time for various lateral pressures (compression). As can be seen, in the range from 1 to +40 bar, the drop rate becomes slower and the plateau shifts up gradually with the increase in lateral pressure, which indicates reduced orientational mobility and restricted motion because of the higher density of the system. Again, and consistently with our previous measurements, an abrupt change in the evolution of the ACF can be observed as the lateral pressure increases from 40 to 50 bar, demonstrating much slower dynamics in the undulated phase compared to the uniform one. As pressure increases further, relaxation becomes progressively slower. This indicates that the lateral pressure also induces a dynamic phase transition, where the orientational mobility of the DPPC molecules suffers a significant drop. A similar study was previously carried out in [53], which showed that the rotational autocorrelation function of the glycerol backbone vector relaxes noticeably slower at the fluid-gel phase transition. A video of a DPPC membrane at 50 bar, provided in the Supplementary Materials, illustrates the phase transition from the uniform phase to a significantly less mobile undulated one. Conformationally, this latter phase corresponds to straight and orientationally aligned acyl chains, as further verified by the trends of the global nematic order parameter and the tilt angle distributions, as presented earlier.

In Figure S1 (Supplementary Materials) we compare the HL and LL simulations. For both, the variation in the APL for the entire pressure range remains very similar, and consequently the uniform-to-undulated phase transition occurs at the same pressure range. It is necessary to mention here that, for HL, the systems near the transition take longer time (more than 600 ns) to reach equilibration compared to LL. Also shown in Figure S1 are the APL trends and the density profiles for HL and LL under compression.

### 3.2. Stretching

**Figure 8:**
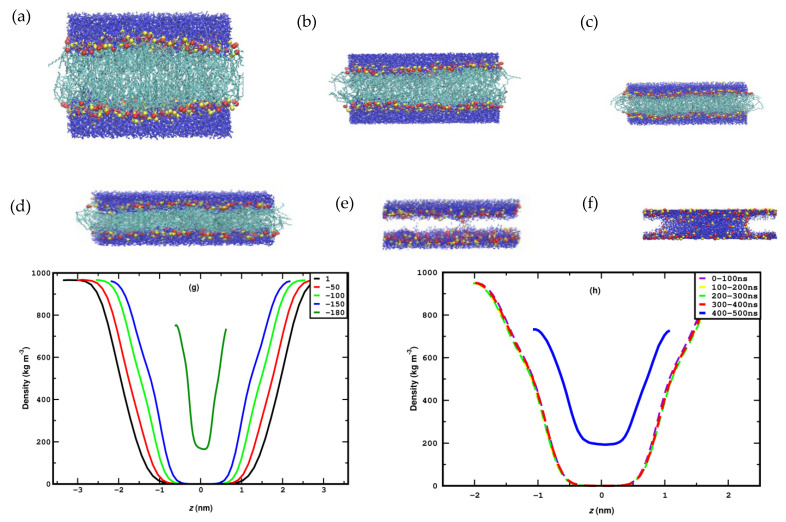
(**a**–**d**) Snapshots of the DPPC membrane during stretching at 1, −50, −100, and −150 bar. Panels (**e**,**f**) focus on the onset (*t* = 104 ns) and completion (*t* = 106 ns), respectively, of the rupture that occurs at −180 bar. In panels (**e**,**f**), the hydrophobic part of the membrane is omitted for visual clarity. The mass density profile of water along the bilayer normal (Z-axis) for different pressures under stretching (panel (**g**)) and for different time intervals for stretching at −180 bar (panel (**h**)) are also shown. The penetration of water into the hydrophobic region leading to rupture is evident at −180 bar.

The effect of stretching on the membrane is also studied between −10 and −200 bar, again in a step of −10 bar, to identify, as accurately as possible, the pressure range where critical phenomena occur. The systems under stretching reach equilibration in roughly 30–50 ns. In systems where the stretching is too high for the membrane to remain stable (−180 to −200 bar), rupture may occur in a time scale between 50 and 500 ns, as discussed in detail later. Hence, the simulations for each stretching value are carried out for 500 ns, and the properties are calculated as averages from the system configurations of the last 350 ns. To directly compare the membrane behavior under stretching and compression, we present the corresponding figures side by side (mass density profile, area per lipid, and the orientational autocorrelation function) or we combine them in the same figure panel (nematic order parameter and carbon tilt angle distribution). Thus, the mass density profile of the lipid headgroup is plotted in Figure 3b, which shows that the separation between the peaks decreases gradually with stretching, indicating membrane thinning which leads to a higher area per lipid. The gradual membrane thinning trend can also be identified visually by the snapshots in Figure 8a–e. For −180 bar stretching, the membrane ruptures, as clearly seen in the snapshots of the panels (f) and (g). Figure 8f represents the snapshot at 104 ns, where a few water molecules can be seen to penetrate the hydrophobic part of the membrane (omitted for clarity). This event practically marks the onset of the membrane rupture. The presence of a water column inside the hydrophobic part, indicating the full rupture of the membrane, is evident in the snapshot of Figure 8g corresponding to *t* = 106 ns. Figure 8h shows the gradual reduction in the width of the hydrophobic region as stretching increases due to the thinning of the membrane. Eventually, at −180 bar, as also confirmed by the density profile, the water molecules percolate between the two layers due to membrane rupture, as further evidenced visually from the snapshot in Figure 8g. Figure 8h shows that at −180 bar, the rupture of the membrane occurs after 400 ns.

In parallel, A_XY_PL increases linearly with stretching, as confirmed by the best fit shown in Figure 3d, suggesting the linear areal expansion of the membrane. As illustrated in Figure 3d, the variance of the head group σ_Z_ exhibits a linear decrease with an increase in stretching, indicating the more confined planar distribution of the headgroups. Notably, in Figure 3c,d, across the entire pressure range from −160 to +40 bar, where the membrane maintains a uniform planar distribution, *σ*_Z_ depends linearly on lateral pressure for both stretching and compression, though with different slopes. Furthermore, it is important to highlight that the slope of *σ*_Z_ changes distinctly for compressive stresses exceeding 40 bar, at which point the membrane undergoes undulation. Accordingly, *σ*_Z_ serves as a potential parameter for detecting the transition from a uniform distribution to an undulated state.

The nematic order parameter *S* decreases almost linearly with stretching, as shown in Figure 7a, since dilution due to stretching leads to more orientational freedom to the acyl chains and consequently the loss of long-range orientational ordering. The alternate carbon tilt angle distribution gradually broadens with stretching, pointing towards higher flexibility in the acyl chains, as shown in Figure 7b. The autocorrelation function ACF is plotted for various negative pressures in Figure 7d. Faster decay for the ACF could be observed gradually for higher stretching values because of the enhanced orientational mobility. In all cases, ACF reaches a plateau region, which shifts towards a lower value as stretching increases. This shift indicates a larger free volume available to the lipid chains, increasing local mobility. It should be noted that the ACF’s behavior for the entire range of stretching is very similar to the compression value until +40 bar, where the latter suffers a phase change, and the rotational dynamics becomes slower and eventually freezes. The variation in APL, order parameter, and the ACF with stretching is systematic until −170 bar, which contrasts to compression where a discontinuity is seen between 40 and 50 bar, corresponding to a uniform-to-undulated phase transition.

As mentioned earlier, the membrane ruptures at −180 bar, a pressure value which is slightly lower than −200 bar, as reported in the previous MD simulation study by Xie et al. [51] on the DPPC membrane using the lipid-Berger [89] force field parameters. In that study, stretching was varied in increments of 50 bar, primarily focusing on the investigation of water pore formation in the membrane, its stability, and the rupture of the membrane, which typically occurs at elevated stretching levels. The present study employs a finer increment of 10 bar, allowing for a more precise analysis of systematic structural changes and the identification of the critical stretching strength associated with rupture. In another simulation study by Leontiadou et al. [50], which focused on the study of the pore formation and its structural analysis, stretching was applied instantly (referred to as high loading (HL) in the present manuscript), and rupture occurred at −200 bar. Tomasini et al. [14] investigated the rupture of the membrane by increasing the surface tension and reported that the rupture of the membrane occurs for surface tension values between 80 and 90 mN/m, which is equivalent to lateral stretching from −150 to −200 bar. Because of the small difference at the critical strength of the stretching for the rupture of the membrane with the aforementioned works, four independent simulations have been additionally performed at each pressure of −170, −180, −190, and −200 bar to check the statistical ambiguity of rupture in the vicinity of the critical value. In each of the four independent simulations, the same initial configuration is used, but different initial velocities are assigned to atoms. For all independent simulations, the total simulation time is set to 500 ns, unless the membrane ruptures earlier. As can be seen in Table 1, at −170 bar, none of the systems shows rupture; at −180 bar, three of the four systems show rupture; whereas at −190 and −200 bar, all simulations lead to membrane rupture. Accordingly, −170 bar is considered the highest limit of structural stability for the membrane, and thus, no independent simulations were conducted at lower pressures. In parallel, our present data suggest that rupture is certain at −200 bar, which agrees with earlier reports. Furthermore, the present study clearly demonstrates that the membrane becomes vulnerable to rupture already from −180 bar onwards, which overlaps with the predicted range by Tomasini et al. [14].

Also, it should be noticed that the time of rupture of the membrane is highly unpredictable, as can be seen for the cases of −180, −190, and −200 bar, where it varies in a broad range from 50 up to almost 500 ns. A video showing the rupture of the membrane at −180 bar is provided in the Supplementary Materials. Furthermore, the comparison of the loading rate effect for stretching shows no significant difference, as verified by the data in Table S1 of Supplementary Materials.

## 4. Pressure Hysteresis

**Figure 9:**
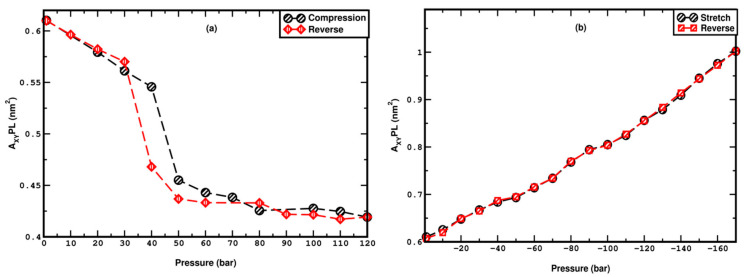
Pressure hysteresis under (**a**) compression and (**b**) stretching. The figures show the variation of A_XY_PL in both compression, decompression, stretching, and relaxation.

The thermal hysteresis associated with the heating and cooling of DPPC has been extensively studied in prior research [88,90]. In the current study, we investigate the hysteresis response of the membrane under pressure. The membrane, initially compressed at 120 bar, is progressively decompressed in a step of 10 bar. Similarly, the membrane that is stretched to −170 bar, which is the highest negative pressure that guarantees structural stability, is relaxed in 10-bar steps. The A_XY_PL metric is employed to quantify the hysteresis trends. As illustrated in Figure 9a, the A_XY_PL during the decompression cycle exhibits sharp increment between 30 and 40 bar, representing an undulated-to-uniform phase transition, which is confirmed by studying the snapshots given in Figure S6 (Supplementary Materials). The overall variation of A_XY_PL in decompression aligns closely with the compression cycle, except that the critical transition pressure is slightly lowered by 10 bar, i.e., between 30 and 40 bar, underscoring the reversible nature of the phase transition. Accordingly, a small hysteresis is observed in the interval between 40 and 50 bar. Conversely, during the stretching cycle, the A_XY_PL decreases gradually, retracing the path of the stretching cycle, further indicating the reversibility of the process and the absence of hysteresis. The snapshots of the change from the undulated to the uniform state for the membrane during the reversible cycle of compression (LL) are shown in Figure S6 (Supplementary Materials).

One important question is how the system size could affect the phase transitions and the critical phenomena (undulation and rupture). To address this, we conduct additional simulations on a smaller system composed of 128 DPPC molecules, as also stated in Section 2. To illustrate the system size effect, for both compression and stretching, the A_XY_PL and mass density plot of headgroups are analyzed. The phase transition for 128 DPPC during compression is found to be at 30–40 bar, slightly lower than the 40–50 bar range for the larger 256-DPPC system. In parallel, the trends observed for stretching appear identical between the two system sizes.

For stretching, A_XY_PL shows excellent agreement at lower pressures (<−80 bar) and a small drift at higher-pressure stretching. The rupture of the membrane occurs at −170 bar compared to −180 bar for the larger system size of 256 DPPC. All aforementioned results are included in Figure S7 in the Supplementary Materials. The system size analysis is performed only for high loading cases. Based on them, we can safely conclude that system size has a reduced effect on the observed trends for the membrane behavior under stretching and compression.

## 5. Conclusions

This study investigates the structural and dynamic behavior of DPPC bilayers under varying lateral pressures for low and high loading rates through atomistic MD simulations, some exceeding 1.5 µs in duration and surpassing any previously published studies by one order of magnitude. We thus identify with high precision a transition from the uniform to the undulated phase occurring at +50 bar, as reflected by independent metrics, such as the autocorrelation function, nematic order parameter, membrane curvature, and headgroup variance along the bilayer normal. This structural alteration is associated with a sharp decrease in the A_XY_PL, an increase in the variance of the headgroups, and a significant reduction in the rotational mobility of the lipid chains. Furthermore, the mean curvature analysis qualitatively demonstrates the undulated conformation of the membrane following this transition. Also, the 3D-APL, calculated using the Delaunay triangulation method, reveals that the conformational transition has a weaker effect on lipid packing density.

The nematic order parameters confirm the absence of cross-tilt configurations of the acyl chains. The tilt angle distribution not only shows the gradually ordering of the acyl chains with compression but also reveals the presence of a ripple phase at higher pressures. Compression shows a slight hysteresis on reversing the pressure and is thus found to be a reversible process. The systems simulated below the phase transition (up to 40 bar) reach equilibrium within a simulation time of 30–50 ns, whereas, above the transition, the equilibration requires a significantly longer time of 400–600 ns. This essentially means that an appreciably longer simulation time is required to study DPPC bilayers under high compression, which could explain why the observed phase transition has not been reported in the past.

The effect of the loading rate is not profound, except near the transition pressure. For stretching, the effect of pressure is much more regular compared to compression. The rotational mobility of the lipid chains also increases with stretching. The tolerance of the membrane against rupture is also examined through multiple independent simulations. It is observed that, independent of the pressure loading pattern, the membrane can withstand a stretching strength up to −170 bar, maintaining its structural integrity, and there is probability of membrane rupture from −180 bar onwards. Membrane rupture becomes certain at −200 bar. Typically, systems equilibrate under stretching within 50 ns. However, under extreme stretching conditions, particularly when the membrane is susceptible to rupture (between −180 to −200 bar), the rupture may take 400–500 ns to occur, emphasizing the necessity of extended simulation times. The effect of loading rates is found to be minimal for the case of stretching, except that the rupture at −200 bar occurs at earlier times for high loading. The system size effect is found to be minimal in the observed phase transition during compression, and the same can be stated for the structural behavior and dynamical response of the DPPC bilayer.

The present findings are important because fluctuations in local membrane pressure can significantly impact membrane function. From the modeling perspective, it is established that critical phenomena and transitions can be observed only through sufficiently long simulation times. Furthermore, this study is expected to stimulate further research, including studies on the localization and dynamic properties of drugs as well as the interaction of small molecules with the membrane, as carried out in many recent works [91,92,93,94,95]. Understanding these aspects is critical for elucidating processes like drug–receptor binding and drug translocation across membranes, which are vital in pharmacological research and drug development.

## Figures and Tables

**Table 1:** Four independent simulations at each pressure for low loading stretching reporting the occurrence of rupture and the corresponding time (when applicable; otherwise, “NA” appears).

Pressure Low Loading (bar)	Simulation Index	Rupture?	Time of Rupture (ns)
−170	#1	No	NA
−170	#2	No	NA
−170	#3	No	NA
−170	#4	No	NA
−180	#1	Yes	106
−180	#2	Yes	426
−180	#3	Yes	416
−180	#4	No	NA
−190	#1	Yes	238
−190	#2	Yes	74
−190	#3	Yes	103
−190	#4	Yes	458
−200	#1	Yes	188
−200	#2	Yes	55
−200	#3	Yes	207
−200	#4	Yes	154

## Data Availability

All raw data from the simulations reported here are available upon request.

## Supplementary Materials

The following supporting information can be downloaded at https://www.mdpi.com/article/10.3390/membranes15060161/s1: Figure S1. Comparison between high loading (HL) and low loading (LL) under compression and stretching. Table S1: Four independent simulations at each pressure for high loading (HL) stretching reporting the occurrence of rupture and the corresponding time. Figure S2. Contour plot of mean curvature (nm^−1^) across the top leaflets of a DPPC 256 bilayer at −180, −150, −100, 1, 40, 50, 70, 80, and 100 bar, projected onto the XY-plane. Figure S3. A coarse-grained sketch of the DPPC layer in different phases. Figure S4. Ripple phase shown at a pressure of 50, 70 and 100 bar corresponding to compression (low loading, LL). Figure S5. Absolute value of the deuterium order parameter of the Sn1 acyl chain of DPPC, |*S*_CD_|, for compression and stretching for low loading. Figure S6. Snapshots showcasing the change from undulated to uniform membrane during the reversible cycle of compression (LL) at 40 and 30 bar. Figure S7: Comparison between the 128-DPPC and 256-DPPC systems for low loading (both compression and stretching). Video showcasing the compression at 50 bar and rupture at −180 bar.
