# Supplementary material for: DPPC Membrane Under Lateral Compression and Stretching to Extreme Limits: Phase Transitions and Rupture
Source: Membranes (Basel). 2025 May 26;15(6):161. doi: 10.3390/membranes15060161 (PMC12194903; doi:10.3390/membranes15060161)
Supplement: Supplementary file 1 [file membranes-15-00161-s001.zip › 3577053-supplementary_pf.pdf]

SUPPLEMENTARY MATERIALS

DPPC Membrane Response Under Varied Lateral Compression and Stretching to Extreme Limits: Phase Transitions and Rupture

*Subhalaxmi Das,[1] Nikos Ch. Karayiannis,[2] Supriya Roy[1] **

[1] School of Applied Sciences, Kalinga Institute of Industrial Technology (KIIT) Deemed to be University, Bhubaneswar, Odisha, India, 751024
[2] Institute for Optoelectronic Systems and Microtechnology (ISOM) and Escuela Técnica Superior de Ingenieros Industriales (ETSII), Universidad Politécnica de Madrid (UPM), José Gutiérrez Abascal 2,E-28006 Madrid, Spain
* Correspondence: author, e-mail: supriyophy163@gmail.com

EFFECT OF LOADING RATES ON COMPRESSION AND STRETCHING:

No significant differences are noticed in the static and dynamic properties of the membrane between the HL and LL pressure modes, as seen in Figure S1. However, in the pressure range close to the phase transition (i.e., 50 bar), the time required for the equilibration of the membrane reaches 1.5 μs in case of HL compression, while the corresponding one is around 500 ns for LL compression.

Apart from this, in the case of HL compression, we cannot simulate beyond +100 bar (165 ns) due to the structural instability of the system, whereas simulations are feasible for up to +150 bar in case of low loading. Contradictory to the present findings, a study carried out by Xie et al. [51] observed slight undulation at 200 bar with a high loading rate. They simulated a 128-DPPC system for 250 ns with an initial structure of APL = 0.656 nm-2, which is a bit higher than the one we use for this study. A more relaxed initial structure may have resulted in increased tolerance for higher compression loads.

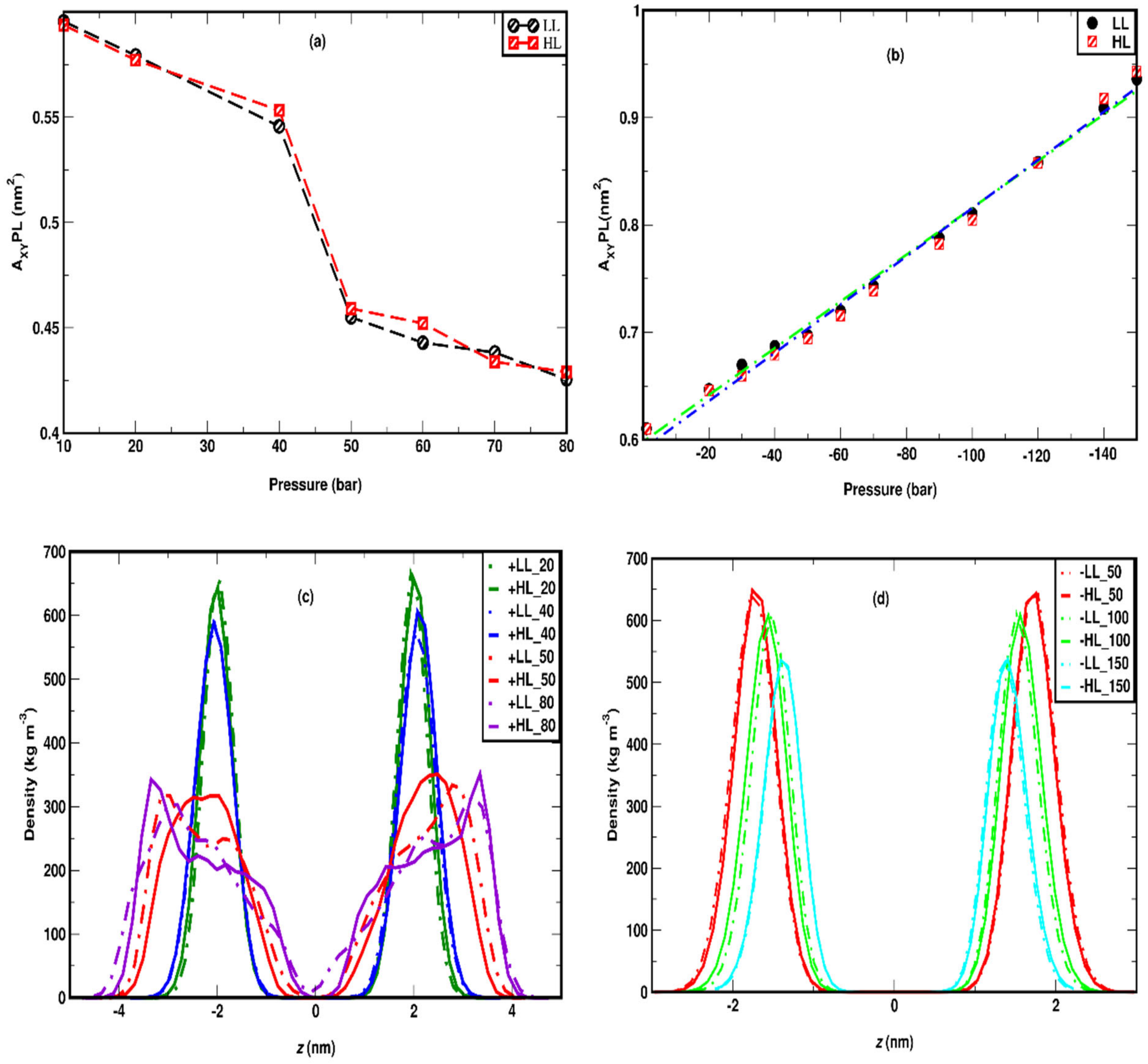

Figure S1. Comparison between high loading (HL) and low loading (LL) under compression: Area per lipid for compression (panel a) and stretching (panel b), respectively. Mass density profile for compression (panel c) and stretching (panel d), respectively.

The results corresponding to LL compression exceeding 100 bar are not included here as the membrane becomes too undulated for analysis. As in the case of LL stretching, four independent simulations at -170, -180, -190, and -200 bar are carried out for HL stretching. All (four) systems rupture at -180, 190, and 200 bar, while none does at -170 bar. Thus, we can conclude that membrane rupture is certain at -180 bar, while -170 bar can be considered the highest applied pressure at which the structural stability of the membrane is guaranteed. The summary of the results, including the time when rupture occurs, is reported in

Table S1. It is observed that the rupture at high loading -200 bar always occurs earlier than at low loading. At -180 and -190 bar, the rupture event becomes certain while there is no prominent effect related to the loading rates.

Table S1: Four independent simulations at each pressure for high loading (HL) stretching reporting the occurrence of rupture and the corresponding time (when applicable; otherwise, "NA" is reported).

**Pressure (bar) High Loading**	**Simulation Index**	**Rupture?**	**Time of Rupture (ns)**
-170	#1	No	NA
-170	#2	No	NA
-170	#3	No	NA
-170	#4	No	NA
-180	#1	Yes	280
-180	#2	Yes	418
-180	#3	Yes	435
-180	#4	Yes	209
-190	#1	Yes	274
-190	#2	Yes	194
-190	#3	Yes	187
-190	#4	Yes	47
-200	#1	Yes	16
-200	#2	Yes	45
-200	#3	Yes	72
-200	#4	Yes	13

MEAN CURVATURE:

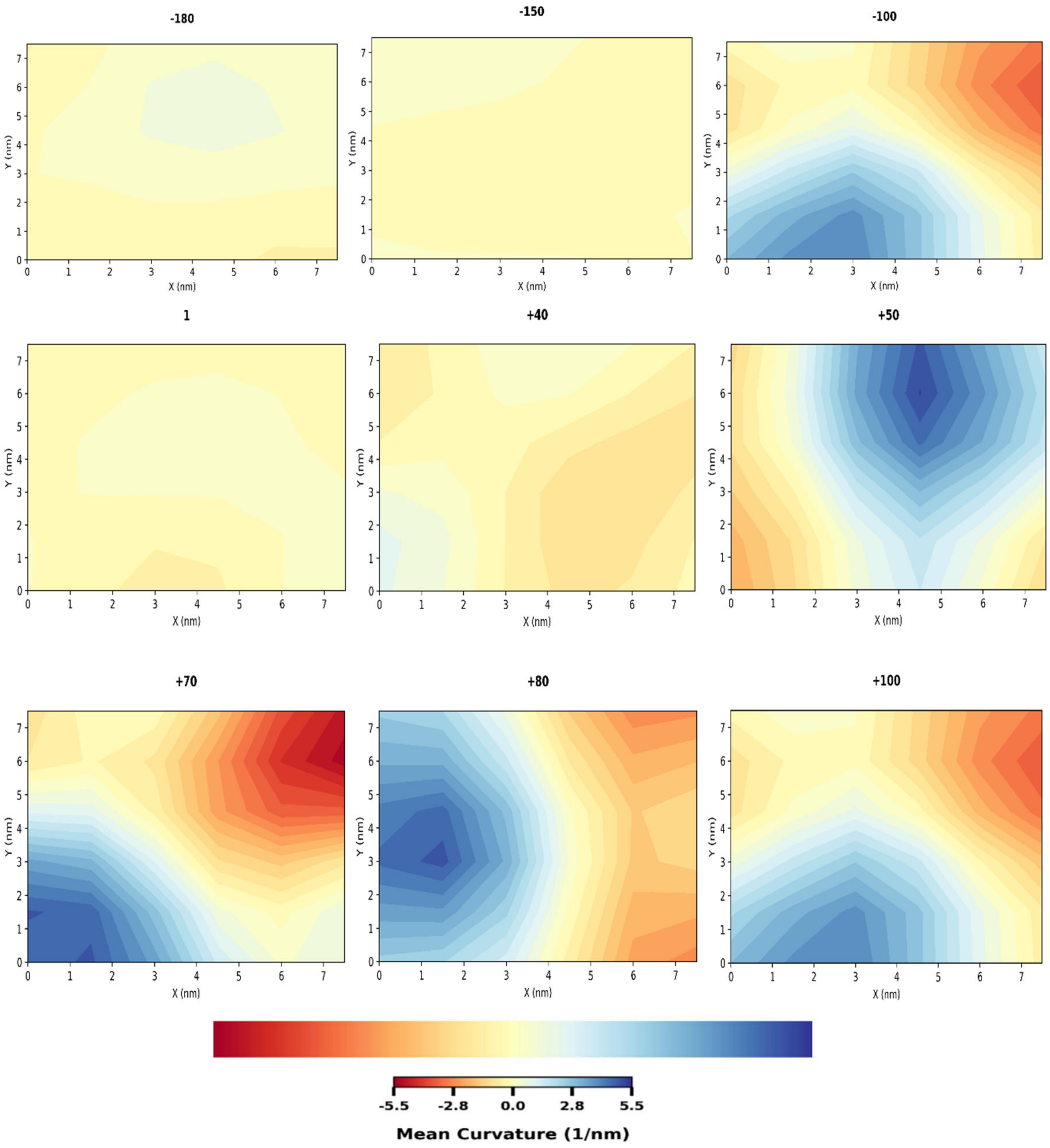

Figure S2. Contour plot of the mean curvature (nm^{-1}) across the top leaflets of a DPPC 256 bilayer at -180, -150, -100, 1, 40, 50, 70, 80, and 100 bar, projected onto the XY-plane. In the presented colormap, blue, yellow, and red correspond to convex, flat, and concave regions, respectively. The bottom leaflet shows similar behavior, hence it is not presented here.

THE PHASES OF THE DPPC MEMBRANE:

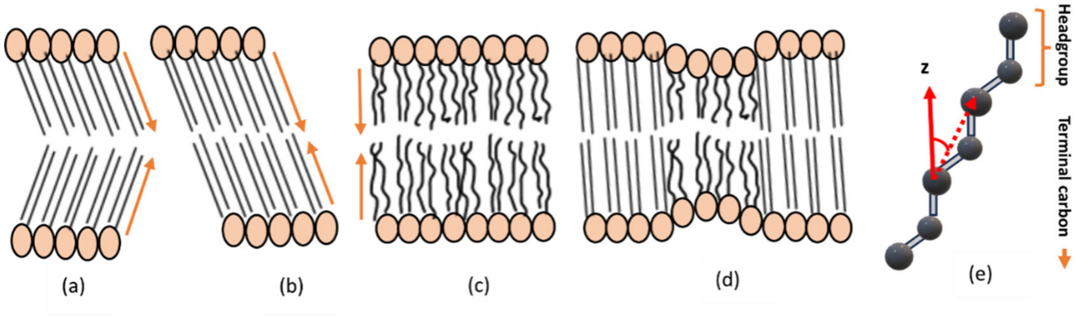

Figure S3. A coarse-grained sketch of the DPPC layer in different phases, similar to the ones presented in[85] and [88]. The orange circle represents the headgroup, and the straight lines represent the acyl chains. The orange arrows correspond to the nematic directors of the top and bottom leaflet. Configurations (a) and (b) correspond to the cross tilt and tilt phases, respectively. The disordered and ripple phases are shown in (c) and (d), respectively. (e) Representation of the vector (in red dashed line) formed by successive carbon units (black spheres) defining an angle with the Z-axis.

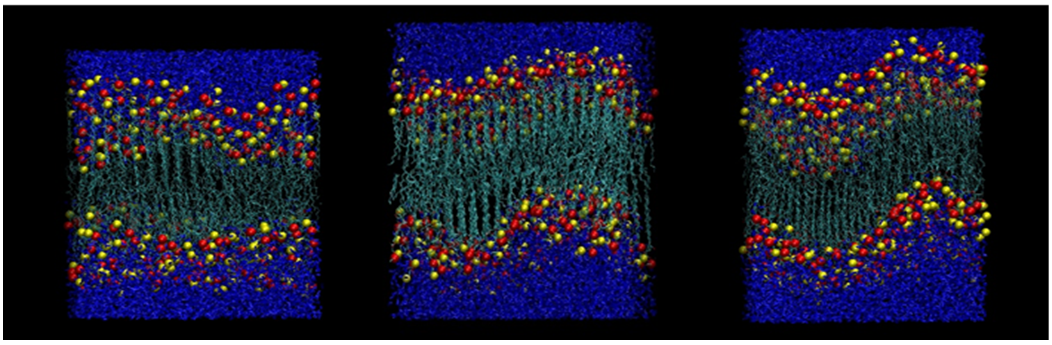

Figure S4. Ripple phase shown at a pressure of 50 (left), 70 (middle), and 100 (right) bar corresponding to compression (low loading, LL). The red and yellow spheres represent the headgroup P and N atoms, respectively. The blue and cyan colors represent the solvent (water) and the acyl chains, respectively. The black background is chosen to make phases appear more prominent.

DEUTERIUM ORDER PARAMETER:

The deuterium order parameter (S_{CD}) quantifies the alignment of C–H bonds relative to the bilayer normal and is commonly used to study phase transitions, such as the gel-to-liquid crystalline transition of bilayer membranes. In the present study, however, the membrane becomes undulated beyond a critical pressure, leading to the development of local curvature and spatial variation in the bilayer normal. As a result, $|S_{CD}|$ cannot be reliably calculated assuming a fixed bilayer normal; instead, it would require the construction of a local normal vector field, which significantly complicates the calculation. However, for reference, we have added the S_{CD} order parameter, calculated with respect to the Z-axis, which is the bilayer normal for the planar membranes (Figure S5). The order parameter increases systematically from 1 to 40 bar and shows a sharp jump between 40 and 50 bar, which is clearly correlated to the

uniform-to-undulated phase transition. In parallel, during stretching, the order parameter systematically decreases, indicating a loss of lipid tail ordering.

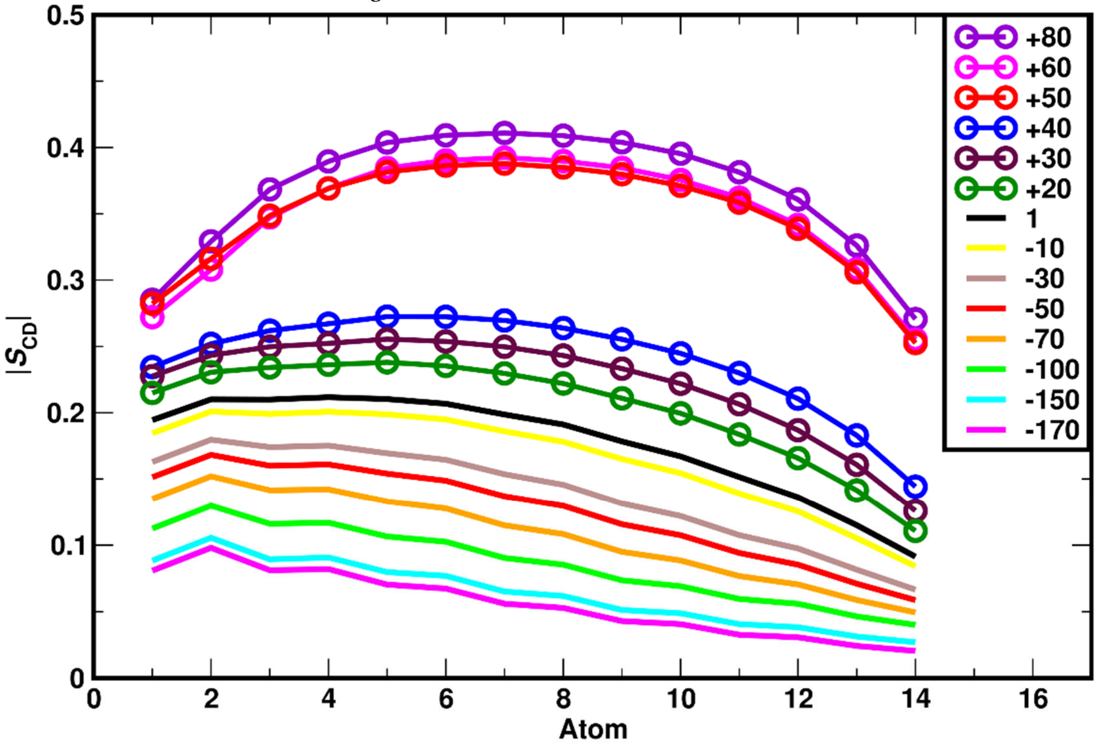

Figure S5. Absolute value of the deuterium order parameter of the Sn1 acyl chain of DPPC, |S_{CD}|, for compression and stretching for low loading.

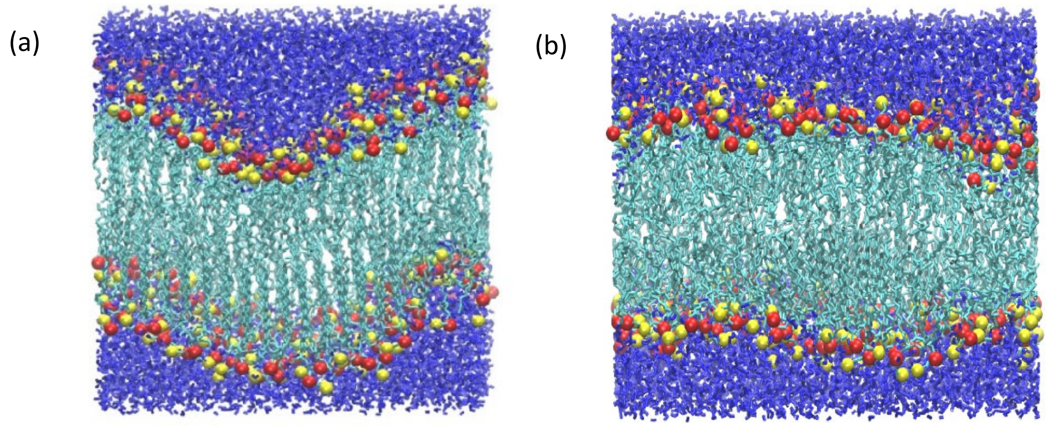

Figure S6. Snapshots showcasing the change from undulated to uniform membrane during the reversible cycle of compression (LL) at 40 (panel a) and 30 bar (panel b).

SYSTEM-SIZE EFFECT:

Simulations of the 128-DPPC system show no significant difference compared to the reference and larger system (256-DPPC molecules) with respect to the structural behavior during compression, as seen in Figure S7. There is a small shift of 10 bar in the phase transition, which happens earlier for the smaller system compared to the larger one. For stretching, there is no detectable effect related to the system size. The variation of $A_{XY}PL$ for low-pressure stretching shows minimal differences between the two systems, while a divergence is observed for higher pressures.

To study rupture under LL stretching, we consider four independent simulations for each pressure around the vicinity of rupture for the 128-DPPC system, as in the case for the larger system. For -170, -180, and -190 bar, rupture occurs in two, three, and all simulations, respectively. No rupture takes place below -170 bar, which also lies within the predicted range.

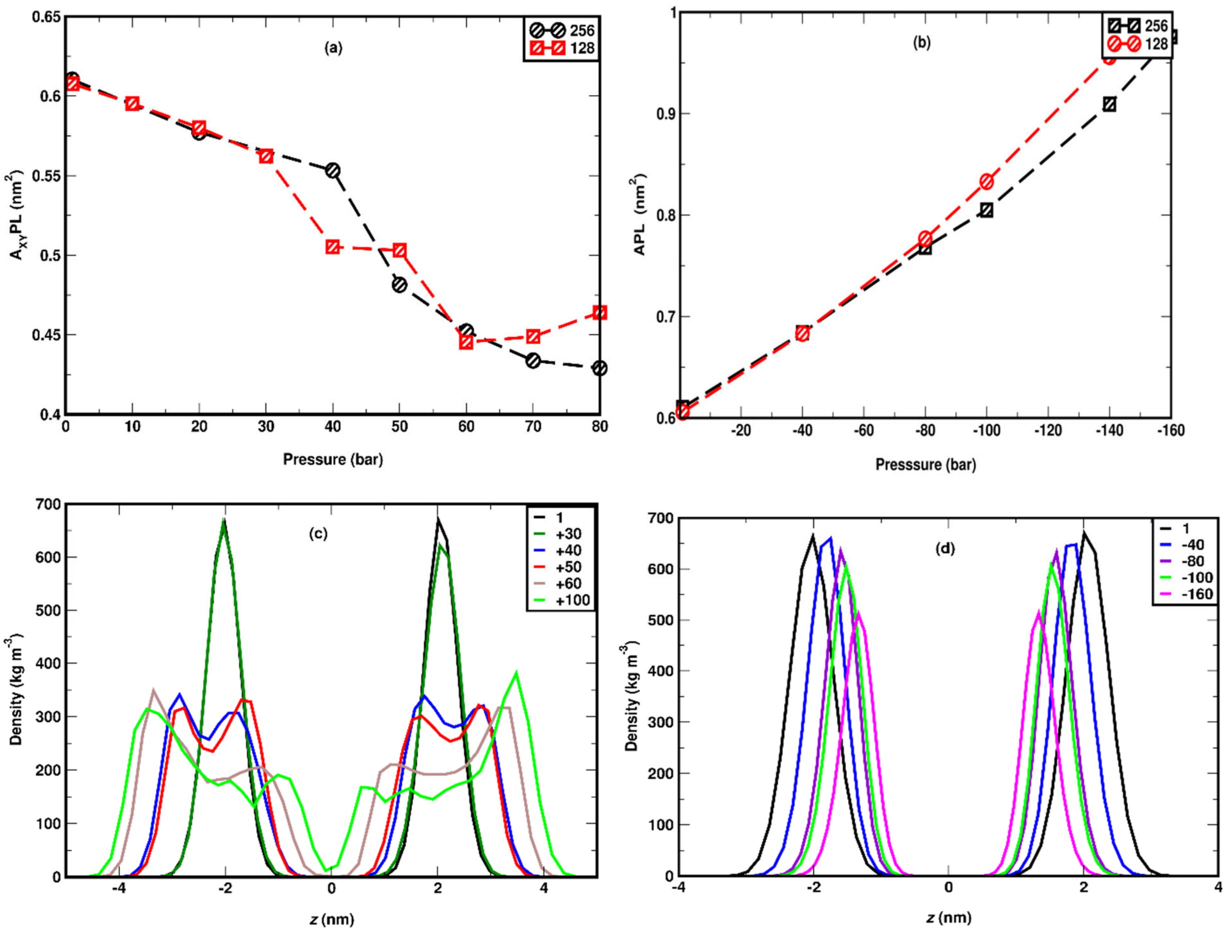

Figure S7: Comparison between the 128-DPPC and 256-DPPC systems for low loading: APL for compression (panel a) and stretching (panel b); mass density profile for compression (panel c) and stretching (panel d).

Videos: Rupture and Undulated State

Video 1 shows how compression changes a membrane from an initially uniform phase to an immobile and undulated one at +50 bar. The lipids are shown in green with the P atom and N atom of the headgroups represented by red and blue spheres, respectively. The water around the membrane is shown by cyan points.

Video 2 shows the gradual rupture leading to the destruction of the membrane at -180 bar. The lipids and water molecules are shown in green and blue, respectively.

References

[51] J. Y. Xie, G. H. Ding, and M. Karttunen, "Molecular dynamics simulations of lipid membranes with lateral force: Rupture and dynamic properties," Biochim Biophys Acta Biomembr, vol. 1838, no. 3, pp. 994–1002, Mar. 2014, doi: 10.1016/j.bbamem.2013.12.011.

[85] S. Leekumjorn and A. K. Sum, "Molecular studies of the gel to liquid-crystalline phase transition for fully hydrated DPPC and DPPE bilayers," Biochimica et Biophysica Acta (BBA) - Biomembranes, vol. 1768, no. 2, pp. 354–365, Feb. 2007, doi: 10.1016/J.BBAMEM.2006.11.003.

[88] S. S. Qin, Z. W. Yu, and Y. X. Yu, "Structural characterization on the gel to liquid-crystal phase transition of fully hydrated DSPC and DSPE bilayers," J Phys Chem B, vol. 113, no. 23, pp. 8114–8123, Jun. 2009, doi: 10.1021/JP808779R.
